# APC/C is essential for hematopoiesis and impaired in aplastic anemia

**DOI:** 10.18632/oncotarget.18808

**Published:** 2017-06-28

**Authors:** Jia Wang, Min-Zhi Yin, Ke-Wen Zhao, Fang Ke, Wen-Jie Jin, Xiao-Lin Guo, Tian-Hui Liu, Xiao-Ye Liu, Hao Gu, Xiao-Min Yu, Zhen Li, Li-Li Mu, Deng-Li Hong, Jing Chen, Guo-Qiang Chen

**Affiliations:** ^1^ Key Laboratory of Cell Differentiation and Apoptosis of Chinese Ministry of Education, Department of Pathophysiology, Shanghai Jiao Tong University School of Medicine (SJTU-SM), Shanghai, 200025, China; ^2^ Department of Pathology, Shanghai Children's Medical Center, SJTU-SM, Shanghai, 200025, China; ^3^ Department of Orthopaedics, Shanghai Ninth People's Hospital, SJTU-SM, Shanghai, 200025, China; ^4^ Key Laboratory of Pediatric Hematology and Oncology Ministry of Health, Department of Hematology and Oncology, Shanghai Children's Medical Center, SJTU-SM, Shanghai, 200127, China

**Keywords:** anaphase promoting complex/cyclosome (APC/C), Anapc2, hematopoietic stem and progenitor cells (HSPCs), dormant HPSCs, aplastic anemia

## Abstract

Anaphase promoting complex/cyclosome (APC/C) is essential for cell cycle progression. Recently, its non-mitotic functions were also reported but less studied in several tissues including hematopoietic cells. Here, we developed an inducible *Anapc2* (a core subunit of APC/C) knockout mice. The animals displayed a fatal bone marrow failure within 7 days after knockout induction. Their hematopoietic stem and progenitor cells (HSPCs) demonstrated a sharp decline and could form little colony. Further, the results of BrdU label-retaining cell assay showed that the dormant HPSCs lost rapidly. Analysis of cell cycle regulators, Skp2, P27, Cdk2, and Cyclin E1, suggested that these quiescent stem cells underwent a shift from quiescence to mitosis followed by apoptosis. We next detected Anapc2-expression in the CD34^+^ HSPCs of patients with aplastic anemia. CD34^+^ cells were markedly decreased in the bone marrow and Anapc2-expression in the residual CD34^+^ cells was undetectable, suggesting that APC/C was deficient and might have a relationship with the pathogenesis of aplastic anemia.

## INTRODUCTION

Hematopoietic stem cells (HSPCs) are tightly regulated by the extrinsic and intrinsic factors [1–5]. Among them various cell-cycle factors such as cyclins and cyclin-dependent kinase inhibitors (CKIs) are important and regulated by Anaphase-promoting complex/cyclosome (APC/C). APC/C is one of important E3 ubiquitin ligase complexes that assemble polyubiquitin chains on specific substrates. It plays distinct roles in the M and G1 phase transiently with two co-activators Cdc20 and Cdh1 [6–8]. In the M phase, Securin and cyclin B, the inhibitors of separase, are degraded by APC/C^Cdc20^, which makes separase activation and cohesin cleaved. Subsequently, mitosis enters into anaphase when Cdh1 replaces Cdc20 to bind with APC/C [6–8]. APC/C^Cdh1^ degrades Cdc20 and other cyclins such as Aurora and Plk1 to advance the cell cycle into the G1 phase [6–8]. In the G1 phase, type A and B cyclins and DNA replication regulators such as Skp2, Geminin, Cdc 6, Tk1 and Tmpk are degraded by APC/C^Cdh1^, guaranteeing a sufficient preparation for entering into the S phase [6–8].

In addition to the role in cell cycle, the cell-cycle-independent functions of APC/C have been recently identified [9–11]. Evidences indicate that the impaired function of APC/C could influence on axon and dendrite growth, lymphocyte metabolism, myogenic fusion of muscle cells and initiation of differentiation of lens [9–11]. In Drosophila embryo, loss of *fzr* leads to extra epidermal cell division [12]. And in adult mice, loss of APC/C in quiescent hepatocytes causes re-entry into cell cycle even without any proliferative stimulus [13]. Although the previous studies showed that APC/C is required for hematopoiesis [13, 14], the underlying cellular and molecular mechanism and its relationship with hematopoietic diseases remains to be investigated. In this study, we developed a mouse model in which the function of APC/C can be conditionally abolished by deleting its core subunit Anapc2 in hematopoietic cells to study the function of this complex in details. We further studied the alteration of Anapc2 in the bone marrow (BM) failure disease, aplastic anemia (AA).

## RESULTS

### Deletion of the Anapc2 allele generates deficient APC/C

**Figure 1:**
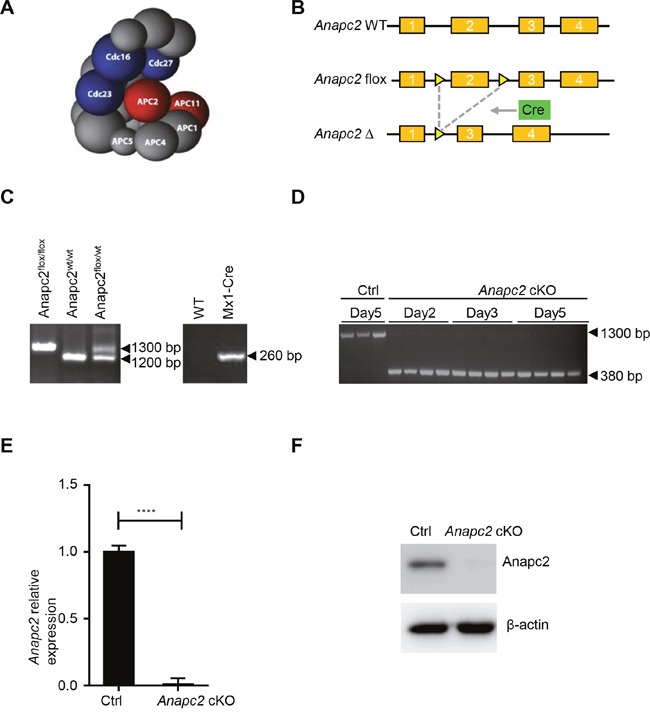
Generation of *Anapc2* conditional knockout (*Anapc2 cKO*) mice (*Anapc2flox/floxMx1*-Cre mice) **(A)** The structure of APC/C [18]. Anapc2 (APC2) and Anapc11 (APC11) (red) comprise the catalytic core and are therefore required for its function. **(B)** Conditionally knocking out *Anapc2* by using LoxP/Cre system. The exon 2 of *Anapc2* was flanked by loxP sites (yellow triangle). After recombination by CRE (green box), exon 2 and one LoxP site were removed. **(C)** Genomic DNA PCR to confirm *Anapc2^flox/flox^* and *Mx1-Cre* allele in *Anapc2^flox/flox^Mx1-Cre* mice after mating *Anapc2^flox/flox^* mice and *Mx1-Cre* mice using peripheral blood genomic DNA. **(D)** Genomic DNA PCR to confirm unexcised (*Anapc2^flox^*) allele (1300 bp) and excised (*Anapc2^Δ^*) allele (380 bp) using BM cells. The *Anapc2* allele was excised after the pIpC injection, results at day 2, 3, 5 are shown. The *Anapc2^flox/flox^* mice were used as control (Ctrl). **(E)** Quantitative PCR to determine relative *Anapc2* mRNA expression level using BM cells. The expression level of *Anapc2* mRNA significantly decreased after the pIpC injection. **** *p*<0.0001. **(F)** Western blot analysis to detect Anapc2 expression using BM cells. The cells used in E and F were from *Anapc2* cKO mice at day 5 post-pIpC injection and *Anapc2^flox/flox^* mice as control (Ctrl).

To create a conditional loss-of-function model for APC/C, Anapc2, one of its core subunits and required for its function [15] (Figure 1A), was conditionally inactivated by a Cre-LoxP system (Figure 1B). In *Anapc2^flox/flox^Mx1-Cre* mice, the exon 2 of *Anapc2* gene which was flanked with LoxP site was excised by Mx1-Cre after the injection of inducer such as pIpC [16] (Figure 1B). The *Anapc2^flox/flox^Mx1-Cre* mice (named *Anapc2* cKO mice) was generated from mating between *Anapc2^flox/flox^* and *Mx1-Cre* mice and their genotyping was confirmed by using general PCR of peripheral blood cells (Figure 1C). Then the genomic PCR, qPCR and WB of BM cells was conducted to assess knockout efficiency of *Anapc2* after the pIpC injection (Figure 1D and 1F). The *Anapc2^flox/flox^* mice were used as control (Ctrl). As expect, the *Anapc2* allele of *Anapc2^flox/flox^Mx1-Cre* mice could be inducibly deleted in the BM (Figure 1D), its *Anapc2* mRNA level of BM cells was significantly decreased (Figure 1E) and subsequently the protein expression level of Anapc2 could not detected (Figure 1F). These data showed that we successfully generated *Apc2Anapc2* conditional knockout (*Anapc2* cKO) mice.

### Anapc2 deletion results in rapid BM failure

**Figure 2:**
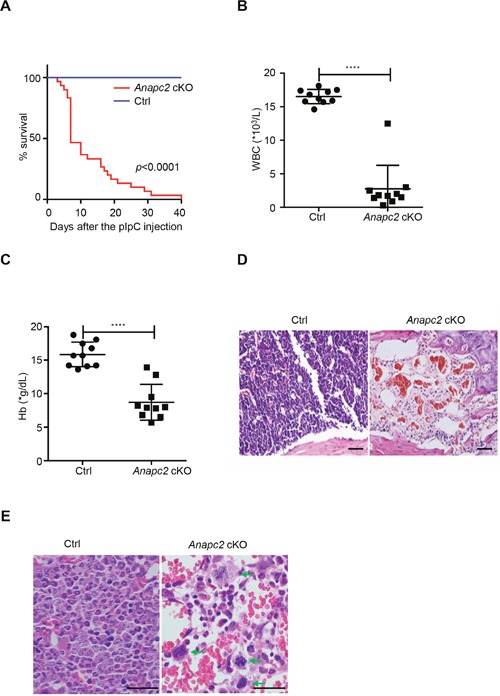
The *Anapc2* deletion resulted in BM failure **(A)** Survival curve of *Anapc2* cKO and *Anapc2^flox/flox^* mice (Ctrl) after the pIpC injection (n=30). Most *Anapc2* cKO mice died within 40 days. (**B** and **C**) Peripheral white blood cell (WBC) and hemoglobin (Hb) counts of *Anapc2* cKO mice significantly decreased at day 5 after the pIpC injection. ****p<0.0001. **(D)** HE staining analysis of BM sections from the femur of *Anapc2* cKO mice and control (Ctrl). After the pIpC injection, most nucleated cells disappeared in the BM. Scale bar represents 100 μm. **(E)** HE staining analysis of BM sections from the femur of *Anapc2* cKO mice and control (Ctrl). An increase of mitotic cells which were arrested in a metaphase-like state (green arrow) was observed in the bone marrow before the disappearance of hematopoietic cells. Scale bar represents 100 μm.

To address the functional role of APC/C on hematopoiesis, we investigated the changes in peripheral blood and BM of *Anapc2* cKO mice after the pIpC injection. At day 3 after the injection, the *Anapc2* cKO mice began to die. At day 7, about half of them died and within a month most died (Figure 2A). The blood routine examination showed that the count of white blood cell (WBC) and hemoglobin (Hb) sharply decreased within 7 days after the injection (Figure 2B and 2C). The histological analysis of BM showed that the hematopoietic tissue dramatically reduced. At day 7, almost all the hematopoietic cells disappeared and only erythrocytes as well as a few lymphocytes existed (Figure 2D). Prior to this end, an increase of mitotic cells was observed in the bone marrow before the disappearance of hematopoietic cells (at day 1 after the pIpC injection) (Figure 2E).

To observe the dynamic changing in the BM, serial histological analysis was performed. From day 1 after the pIpC injection until the point at which about half of the mice died (at day 7), the histological change of BM was successively observed. Interestingly, the disappearance of hematopoietic cells showed some characteristics. The HE of femur showed that the hematopoietic cells began to reduce from the metaphysis shortly at day 1 and gradually the reduction developed to the diaphysis. At the day 7, the majority of hematopoietic cells disappeared and only in the diaphysis exited very few nucleated cells (Supplementary Figure 1 and 2). Considering that the hematopoietic stem and progenitor cells (HSPCs) enrich in the metaphysis, the characteristic spatial pattern of hematopoietic cell disappearance suggested that the HSPCs decreased at the early stage and nearly completely disappeared in a short time.

### The hematopoietic stem and progenitor cells cannot be maintained after Anapc2 deletion

**Figure 3:**
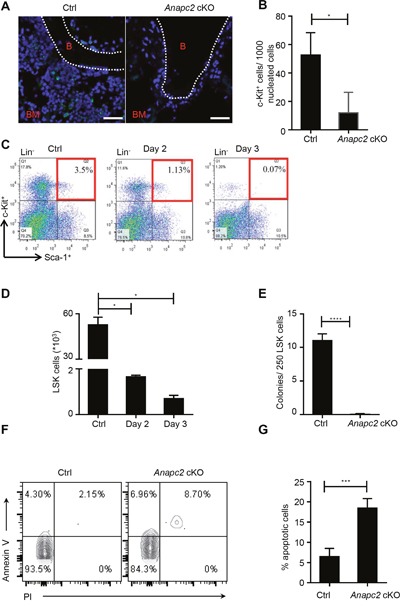
*Anapc2* is required for the maintenance of HSPCs (**A** and **B**) The c-Kit^+^ cells from the femur of *Anapc2* cKO mice significantly decreased at day 5 after the pIpC injection by using IF staining. c-Kit staining (green), DAPI (blue), B (bone), BM (bone marrow), n=5, scale bar represents 100 μm, * *p*<0.05. **(C)** Flow cytometric analysis of LSK (Lin^−^Sca-1^+^c-Kit^+^) cells from *Anapc2* cKO mice at day 2 and day 3 post-pIpC injection showed LSK cells significantly decreased. **(D)** Absolute number of LSK cells were calculated based on the proportion obtained in Fig. C. n=5, *p<0.01. **(E)** The colony-formation cell (CFC) assay demonstrated that the LSK cells obtained from day 2 post-pIpC injection could hardly generate any colony, n=5, ****p<0.0001. (**F** and **G**) The proportion of Annexin V^+^ LSK cells were significantly increased at day 1-3 after the pIpC injection, n=9, ****p<0.0001.

The histological data showed that the HSPCs decreased at the early stage in a rapid BM failure process after *Anapc2* deletion, so we suspected that these cells were affected. To verify the change of HSPCs, these cells were examined. The c-Kit^+^ cells significantly reduced at day 5 after the pIpC injection by using IF staining (Figure 3A and 3B). The flow cytometry analysis demonstrated that the proportion (Figure 3C) and the accordingly calculated absolute number (Figure 3D) of LSK cells dropped precipitously at day 2 and could be hardly detected at day 3. These data showed a sharp decline of HSPCs.

Next, the colonogenic capacity of these cells was tested by the CFC assay. As expect, the result demonstrated that the LSK cells obtained at day 2 could hardly generate any colony (Figure 3E), suggesting that these cells almost lost their colonogenic capacity after *Anapc2* deletion and had a cell-intrinsic defect.

The apoptosis assay was conducted to determine whether the LSK cells undergo programmed cell death after *Anapc2* deletion by using Annexin V and PI double staining. The result showed that the proportion of Annexin V^+^ LSK cells were significantly increased at day 3 after the pIpC injection (Figure 3F and 3G), indicating that the sharp decline of HSPCs resulted from programmed cell death.

**Figure 4:**
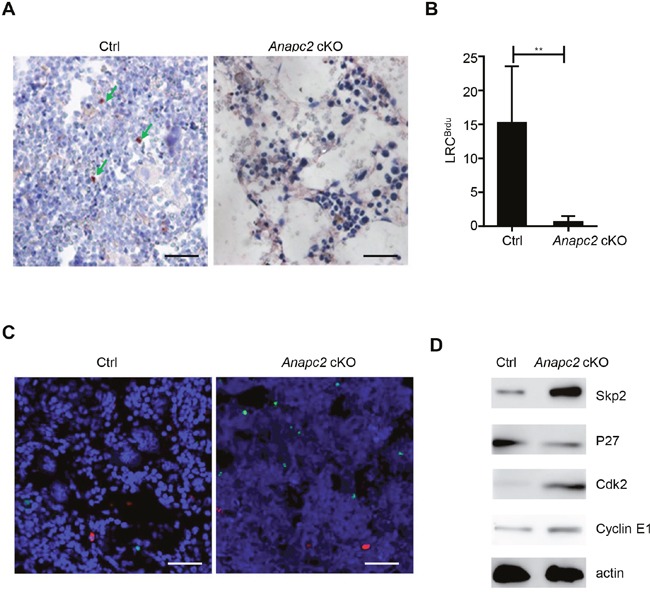
*Anapc2* is required for the maintenance of d-HSCs **(A** and **B)** The LRC^BrdU^ (green arrow) from femur sections of *Anapc2* cKO mice could be hardly detected by IHC staining after the pIpC injection, n=6, ** *p*<0.01. LRC^BrdU^, BrdU label-retaining cells; Ctrl, control. **(C)** None of LRCBrdU showed TUNEL positive in both of Anapc2 cKO mice and control (Ctrl) at day 2 after the pIpC injection, n=6, Anapc2 flox/flox mice as control. LRCBrdU staining (red), TUNEL staining (green), DAPI (blue). Scale bar represents 100 μm. **(D)** Western blot analysis to detect the expression of p27, Skp2, and Cdk2 and cyclin E using BM cells at day 3 post-pIpC injection and *Anapc2^flox/flox^* mice as control (Ctrl).

In the BM, loss of functional HPSCs would be supplied by a small population of reserved d-HSCs, so we then analyzed the change of d-HSCs. Because the d-HSCs have not possessed specific markers, we assessed the morphological change of BrdU label-retaining cells (LRC^BrdU^) in the BM after *Anapc2* deletion. As shown in Figure 4A and 4B, almost all LRC^BrdU^ in the BM markedly reduced and even disappeared in a short time (at day 5∼7) after *Anapc2* deletion, suggesting APC/C may be related with the maintenance of quiescent state of d-HSCs. The TUNEL (TdT-mediated dUTP nick end labeling) assay was conducted with these cells by using BrdU/TUNEL double staining. Unlike the cell population tested above, none of LRC^BrdU^ showed TUNEL positive at day3 after the pIpC injection (Figure 4C), indicated that programmed cell death did not directly account for the disappearance of d-HSCs.

As expected APC/C may a key role in restricting cell cycle entry [13], the cell cycle regulators were analyzed in the hematopoietic cells of our deficient-APC/C-function mice by using western blot (Figure 4D). The cyclin kinase inhibitor p27 was decreased. Meanwhile, the Skp2, which is the substrate of APC/C and degrades p27 [17–19], was increased. And the Cdk2 and cyclin E, inhibited by p27 [20], were increased (Figure 4D). These results indicated that the “threshold” for cell cycle entry was lowered in the hematopoietic cells of our *Anapc2* cKO mice. Together a shift from quiescence to mitosis followed by programmed cell death might result in the disappearance of d-HSCs.

### Anapc2 could not be detected in HSCs of AA

**Figure 5:**
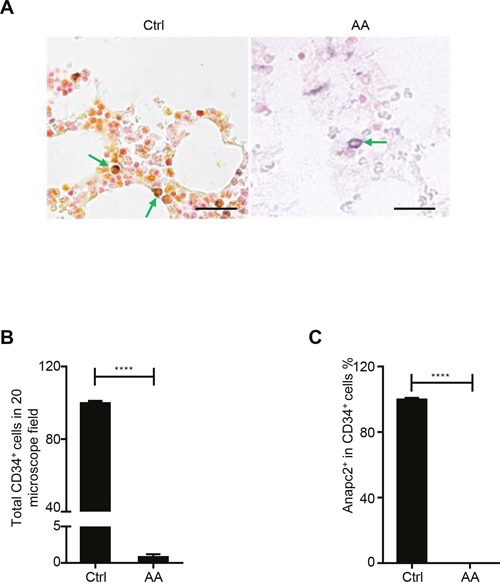
*Anapc2* was absent in the HSPCs in the AA's BM **(A-C)** Anapc2 could not be detected in the BM CD34^+^ cells of AA by bi-color IHC staining. In normal control (Ctrl), Anapc2 (expressed in nuclear, golden in color) could be detected in the BM CD34^+^ (expressed in membrane, blue in color) cells by bi-color IHC staining (green arrow) whereas it could not in AA's (green arrow). n=3 (Ctrl), 20 (AA), scale bar represents 100 μm, ****p<0.0001.

Because the murine BM showed rapid BM failure and a sharp decrease in the number of HSPCs after *Anapc2* deletion, which was similar to the alterations in BM failure diseases, we suspected that impairment of Anapc2 may be related with these diseases such as AA. To investigate it, the CD34/Anapc2 bi-color IHC staining was performed in the BM section of AA patients (Supplementary Table 1) to detect the expression of Anapc2 in the human HSPCs (CD34^+^ cells) in AA. As expected, the numbers of CD34^+^ cells were markedly decreased in AA marrows compared with that in normal controls (Figure 5A and 5B). Interestingly, the residual CD34^+^ cells in the AA marrows did not express Anapc2 whereas the normal group did (Figure 5C). Thus, the Anapc2 may be absent or expressed at an undetectable level in the HSPCs of AA, suggesting that APC/C was deficient and might have a relationship with the pathogenesis of AA.

## DISCUSSION

The function of APC/C in various system such as nervous system has been reported recently [21–24]. In this study, we investigate the role of APC/C in the hematopoietic system through an APC/C-deficient mouse model in which *Anapc2*, the gene encoding the essential APC/C subunit Anapc2, is conditionally deleted. Consistent with the previous study [13], almost all mice in this study demonstrate a decreased peripheral blood cells counts as well as rapid and severe BM failure after *Anapc2* deletion. The LSK cell population was decreased greatly and their colonogenic capacity was almost lost shortly after *Anapc2* deletion. Additionally, a metaphase arrest and programmed cell death was detected in HSPCs. Furthermore, the d-HSPCs disappeared in a short time after *Anapc2* deletion. This may be associated with the function of p27 which directly stabilizes G1/G0 phase of HSCs. Because it was decreased caused by an increased level of Skp2 after *Anapc2* deletion, thereby resulting in an accumulation of cycle E and Cdk2. We thus proposed a model that a shift from quiescence to mitosis of d-HSPCs followed by programmed cell death might cause bone marrow failure.

The phenotype of bone marrow failure caused by a significant decrease of HSPCs in our APC/C-deficient mice share similar characteristics with BM failure diseases such as AA [25–28]. Interestingly, we found that CD34^+^ cells were markedly reduced in the AA BM, and none of the remaining CD34^+^ cells express Anapc2. These results indicated that APC/C might be deficient in these cells and the impaired complex might be related with the pathogenesis of AA. In the future, further studies will be required to figure out all the alterations within the pathway of APC/C in AA cells by using single cell techniques, the dynamics of the alterations in development of the disease, and its prognostic relevance in different subtypes of the disease.

In conclusion, it is the first study to demonstrate the function of APC/C on hematopoiesis and the relationship of this complex with hematological diseases. APC/C was found to be essential for the maintenance of hematopoiesis by helping d-HSCs keep quiescence. And APC/C was found impaired with non-expression of its core subunit-Anapc2 in HSPCs in the AA marrow which might be involved in the pathogenesis of AA. Further insight into the underlying mechanisms would facilitate the exploration of potent preventive and therapeutic methods of AA and other BM failure diseases.

## MATERIALS AND METHODS

### Mice

To produce homozygous *Anapc2* floxed (*Anapc2^flox/flox^*) *Mx1-Cre* strains, *Anapc2^flox/flox^* strains (obtained from Dr. He, Harvard University) were crossed to the *Mx1-Cre* trains (B6.Cg-Tg (Mx1-Cre) 1Cgn/J strains, Jackson Labs). Genotyping of these strains was performed by using genomic PCR with the primers obtained from the providers (primers’ sequences see below). The mice with 6-12 weeks of age were chosen for analysis in this study. All mice were kept under specific pathogen-free conditions in compliance with the National Institutes of Health Guide for the Care and Use of Laboratory Animals with the approval (SYXK-2003-0026) of the Scientific Investigation Board of Shanghai Jiao Tong University School of Medicine, Shanghai, China. For expression of Mx1-Cre, 400 μl pIpC (1 mg/ml in saline, Pharmacia) were injected i.p. twice with an interval of 24 hr.

### Patient bone marrow (BM) samples

Thirty aplastic anemia (AA) patients from Shanghai Children's Medical Center affiliated to Shanghai Jiao Tong University School of Medicine were retrospectively studied. The pathologic diagnosis was conducted by at least two pathologists. Their clinical data and Bouin's fluid-fixed, paraffin-embedded BM sections were obtained with written consent. The normal BM samples were obtained from Shanghai No.9 Hospital affiliated to Shanghai Jiao Tong University School of Medicine. All studies were approved by the Medical Ethical Committee of these hospitals.

### Histopathology

For making murine BM paraffin sections, the femur was collected and placed in Bouin's fixative overnight at room temperature (RT). Other murine tissues were fixed in 10% neutral-buffered formalin. For making frozen section, fresh tissue was embedded in Tissue-Tek O.C.T. Compound (Sakura Finetek) immediately after removed. The 4 μm thick slides were cut for hematoxylin-and-eosin staining (HE) and immunohistochemical (IHC) staining, and the 7 μm thick slides for immunofluorescent (IF) staining. HE staining was performed according to standard procedure (Sigma).

### Immunoassay

The deparaffinization and hydration of 40 μm thick slides was performed as HE staining. The 5-bromo-2-deoxyuridine (BrdU) (1:500 in phosphate buffered saline pH7.4 containing 1% BSA and 2% FCS, Sigma) IHC staining was conducted according to the manufacturer's instructions (Sigma). The c-Kit IF staining was firstly incubated with anti-c-Kit-antibody (1:50, eBioscience) at 4°C overnight after antigen retrieval with citrated buffer pH 6.0 at 98°C for 12min and blocking non-specific binding sites with 5% normal goat serum (Maixin) at RT for 15 min. The next day the slides were incubated with Alexa Flour® 488 goat anti-mouse IgG (H+L) (Maibio) at RM for 1hr. Finally, the slides were counterstained with DAPI (Life) at RM for 15min.

For BrdU/TUNEL double staining, the slides were subjected to dewax, rehydrate and pretreat (denature and trypsin retrieval) as BrdU IHC staining. Then the slides were first incubated with TUNEL reaction mixture (Roche) at 37°C for 1hr, followed by incubation with anti-BrdU-antibody (1:500, Sigma) at 4°C overnight after rinsed with PBS and blocking non-specific binding sites as described above. The next day the slides were incubated with Alexa Flour® 555 goat anti-mouse IgG (H+L) (Maibio) at RM for 1hr. Finally, the slides were counterstained with DAPI (Life) at RM for 15min.

The bi-color CD34 Anapc2 IHC staining was firstly incubated with anti-CD34-antibody (ready-to-use reagent, Mindary) at 4°C overnight after blocking endogenous peroxidase activity with 3% H_2_O_2_ at 37°C for 10 min, antigen retrieval with citrated buffer pH 6.0 at 98°C for 12min and blocking non-specific binding sites with 5% normal goat serum (Maixin) at RT for 15 min. The next day, the slides were incubated with horseradish peroxidase (HRP)-Polymer anti-Mouse IHC Kit (Maixin) at RM for 15 min and proceeded with DAB Kit (blue) (Boster) until the satisfied results were shown under a light microscope. After rinsed with PBS, the slides were incubated with anti-Anapc2-antibody (1:200 in phosphate buffered saline pH 7.4 containing 1% BSA and 2% FCS, Santa Cruz) at 4°C overnight. Subsequently, the slides were incubated with HRP-Polymer anti-Rabbit IHC Kit (Maixin) and proceeded with HighDef^®^ yellow IHC chromogen (HRP) (ENZO) until the satisfied results were shown under a light microscope. Finally, the slides were counterstained with nuclear fast red (Maixin).

All the IHC and IF staining slides were examined by at least 2 investigators.

### Detection of dormant-HSCs (d-HSCs)

The detection of d-HSCs was performed as previously described [29]. For BrdU treatment, mice were injected i.p. only once with 200 μL BrdU (1.8 mg/mL in saline, Sigma) before they drank water containing BrdU (800 μg/mL in ddH_2_O containing 5% glucose, Sigma) for ten days [29]. After 130 days, the retaining BrdU positive cells (LRC^BrdU^) detected by using IHC staining in the BM were regarded as d-HSCs [29, 30].

### Western blot (WB)

Tissue/cell protein lysates were generated by SDS (1%, m/v) lysis buffer. These lysates were equally loaded on a 5% and 10% gel, electrophoresed and transferred to enhanced cheiluminescence-nitrocellulose membranes (Amersham). For detection of protein, the membranes were incubated with antibodies against β-actin (1:10000, Merck-Millipore), Anapc2 (1:1000, Santa Cruz), CyclinB1 (1:1000, Santa Cruz), Cdc20 (1:1000, Santa Cruz), p27 (1:500, Cell Signaling Technology), Skp2(1:1000, Abcam), Cdk2(1:1000, Abways), cyclin E(1:500, Abways), p21(1:300, Santa Cruz), at 4°C overnight, followed by incubation with HRP-conjugated secondary antibody (1:20000, Cell Signaling Technology) at RM for 1 h. The detection of protein signal was performed by a SuperSignal West Pico Chemiluminescent Substrate Kit (Pierce, Rockford).

### Polymerase chain reaction (PCR)

Genotyping of the mice was performed by using general PCR. The genomic DNA of mice was extracted from murine peripheral blood cells according to the manufacture's instruction (TransGen). The PCR was performed according to the manufacture's instruction (TaKaRa) in a 25 μL PCR reaction system with the primers of *Anapc2*-F1-(5′-GCGACAATTATTGCCTCCGATGACTGCGAC-3′), *Anapc2*-R1 (5′-TGGAGAACCCACAACACACATCTGTCCCTTACC-3′), *Cre*-F (5′-GCGGTCTGGCAGTAAAACTATC-3′) and *Cre*-R (5′-AGCAATCCCCAGAAATGCCAG-3′).

Quantitative PCR (qPCR) was performed to confirm expression level of *Anapc2* mRNA with the primers of *Anapc2*-F2 (5′TATGTTGCGCGGAGTCTTGTT-3′), *Anapc2*-R2 (5′-GAAGCACCCATACAGACGCTG-3′). The β-actin was served as external control. Total RNA was purified with TRIzol^®^ Reagent (Invitrogen) as recommended by the manufacturer. After extraction, the extracted RNA was converted into cDNA with cDNA Synthesis Kit (TaKaRa). With these cDNA samples, the quantitative real-time PCR was assayed by using SYBR Green incorporation (Roche). The values of the target gene expression level were normalized to β-actin and were calculated relative to the expression level in control samples. The relative folds were presented as mean ± SEM in three independent experiments with triplicate samples.

### Flow cytometry and colony-formation cell (CFC) assay

BM cell suspensions were prepared from the femur whose cavity was washed and then crushed for cell collection. The LSK (Lin^−^Sca-1^+^c-Kit^+^) cells were prepared by labeling BM lineage^−^ cells suspensions obtained by magnetic bead (STEMCELL) with a mixture of monoclonal antibodies against Sca-1 (1:200, eBioscience), c-Kit (1:200, eBioscience), CD11b (1:400, eBioscience), Gr-1 (1:400, eBioscience), B220 (1:200, eBioscience), CD3 (1:200, eBioscience), TER-119 (1:200, eBioscience), Annexin V (1:50, BD), Propidium Iodide (PI, 1:200, BD). Fluorescence-activated cell analysis was performed on an LSRFortessa cell analyzer (Becton Dickinson). Fluorescence-activated cell sorting was performed on a FACSAriaII Flow Cytometer (Becton Dickinson). Data were analyzed by using FlowJo™ (Tree Star).

CFC was conducted as previously described [31]. It was performed in MethoCult GF M3434 (STEMCELL) containing with 600∼700 LSK cells. After incubating the cells for 14 days, the scored for colony formation was conducted.

### Statistics

Kaplan-Meier survival analysis was used to compare the survival of *Anapc2* conditional knockout (*Anapc2^flox/flox^Mx1-Cre*) and control (*Anapc2^flox/flox^*) mice. Quantitative data was presented as mean ± SEM and analyzed by unpaired student's t-test. Statistical significance was considered as P<0.05. All analyses were conducted by using GraphPad Prism 5.0 (GraphPad Software).

## SUPPLEMENTARY MATERIALS FIGURES AND TABLE


