# Supplementary material for: APC/C is essential for hematopoiesis and impaired in aplastic anemia
Source: Oncotarget. 2017 Jun 28;8(38):63360–9. doi: 10.18632/oncotarget.18808 (PMC5609928; doi:10.18632/oncotarget.18808)
Supplement: Supplementary file 1 [file oncotarget-08-63360-s001.pdf]

APC/C is essential for hematopoiesis and impaired in aplastic anemia

SUPPLEMENTARY MATERIALS

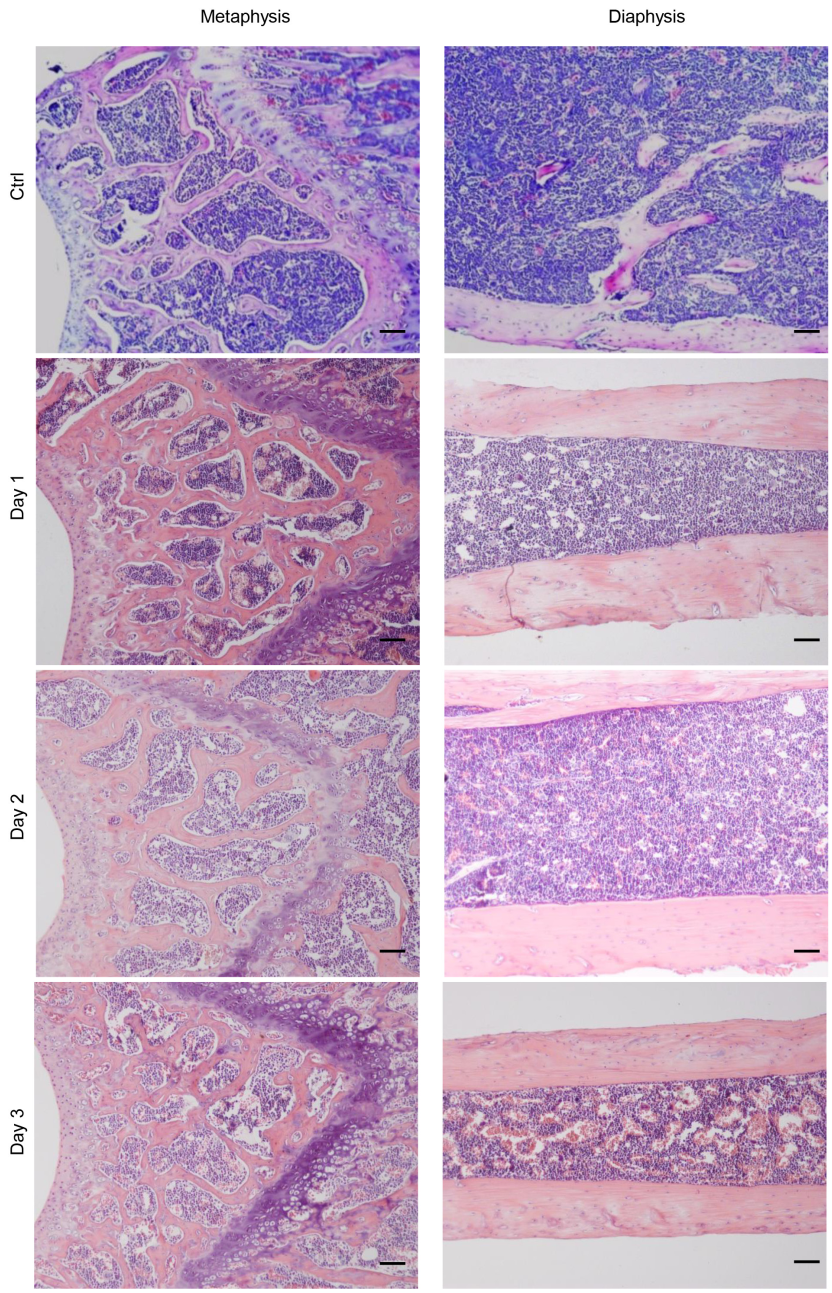

Supplementary Figure 1: Dynamic histological analysis of the femur from *Anapc2* cKO mice and control mice from day 1 to day 7 after the pIpC injection. From day 1, the hematopoietic cells began to reduce from the metaphysis and gradually to the diaphysis. At the day 7, very few nucleated cells were seen only in the diaphysis. Scale bar represent 100 µm. Ctrl, control.

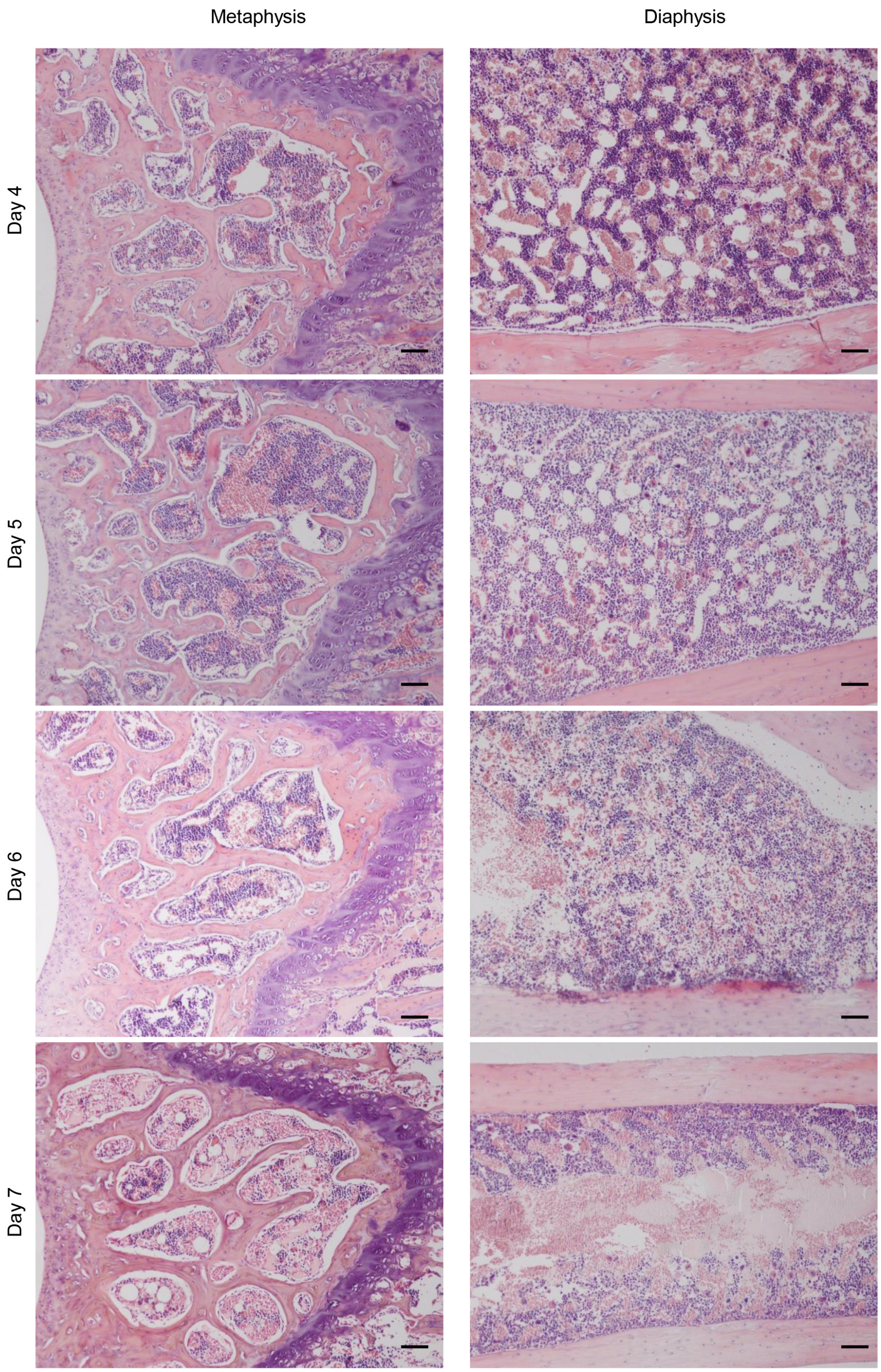

Supplementary Figure 2: Dynamic histological analysis of the femur from *Anapc2* cKO mice and control mice from day 1 to day 7 after the pIpC injection. From day 1, the hematopoietic cells began to reduce from the metaphysis and gradually to the diaphysis. At the day 7, very few nucleated cells were seen only in the diaphysis. Scale bar represent 100 µm. Ctrl, control.

Supplementary Table 1: Characteristics of AA patients

Characteristics	Total (n=30)
Median age (range) at diagnosis, months	55 (7~156)
Sex	
Male (%)	18 (60.0)
Female (%)	12 (40.0)
Severity of AA	
VSAA (%)	5 (16.7)
SAA (%)	24 (80)
NSAA (%)	1(3.3)

AA: aplastic anemia, VSAA: very severe aplastic anemia, SAA: severe aplastic anemia, NSAA: non-severe aplastic anemia.
